# Association of Depression With Atrial Fibrillation in South Korean Adults

**DOI:** 10.1001/jamanetworkopen.2021.41772

**Published:** 2022-01-04

**Authors:** Yun Gi Kim, Kwang-No Lee, Kyung-Do Han, Kyu-Man Han, Kyongjin Min, Ha Young Choi, Yun Young Choi, Jaemin Shim, Jong-Il Choi, Young-Hoon Kim

**Affiliations:** 1 Division of Cardiology, Department of Internal Medicine, Korea University College of Medicine, Korea University Anam Hospital, Seoul, Republic of Korea; 2 Division of Cardiology, Department of Internal Medicine, Ajou University College of Medicine, Suwon, Republic of Korea; 3 Department of Statistics and Actuarial Science, Soongsil University, Seoul, Republic of Korea; 4 Department of Psychiatry, Korea University College of Medicine, Korea University Anam Hospital, Seoul, Republic of Korea

## Abstract

**Importance:**

The risk of atrial fibrillation (AF) in people with depression is not fully known. Depression is associated with sympathetic activation and emotional stress, which might increase the risk of new-onset AF.

**Objective:**

To assess the incidence of new-onset AF in those with and without depression using data from a nationwide health care database.

**Design, Setting, and Participants:**

This cohort study obtained data from the Korean National Health Insurance Service database and enrolled people who underwent a nationwide health checkup in 2009. People younger than 20 years and those with a history of heart valve surgery, previous diagnosis of mitral stenosis, or who were diagnosed with AF between January 1, 2002 and December 31, 2008 were excluded. The risk of new-onset AF (occurring between 2009 and 2018) was compared in people who were and were not diagnosed with depression within a year before the 2009 nationwide health checkup. Data were analyzed between August 1, 2020 and October 31, 2020.

**Exposure:**

Previous diagnosis of depression.

**Main Outcomes and Measures:**

Cumulative incidence and risk of new-onset AF between 2009 and 2018 in participants with and without depression. Kaplan-Meier analysis was conducted to assess incidence of AF, and Cox proportional hazards regression was used to calculate adjusted and unadjusted hazard ratios (HRs) and 95% CIs.

**Results:**

A total of 5 031 222 individuals with a mean (SD) age of 46.99 (14.06) years (2 771 785 men [55.1%]) were included in the analysis; of these individuals, 148 882 (3.0%) had a diagnosis of depression in the year before the 2009 health checkup and 4 882 340 (97%) did not. People with depression vs those without depression were older (aged 56.7 vs 46.7 years) and more likely to be women (96 472 [64.8%] vs 2 162 965 [44.3%]). Prevalence of hypertension, diabetes, dyslipidemia, and heart failure was higher in the depression group. The cumulative incidence of new-onset AF was significantly higher in people with depression vs without depression in the Kaplan-Meier analysis and showed steady divergence throughout 10 years of follow-up (cumulative incidence, 4.44% vs 1.92%; log-rank *P* < .001). After adjusting for covariates, depression was associated with a 25.1% increased risk of new-onset AF (HR, 1.25; 95% CI, 1.22-1.29; *P* < .001). People with recurrent episodes of depression showed even higher risk of new-onset AF (HR, 1.32; 95% CI, 1.27-1.37; *P* < .001). Young age and female sex had significant interactions with depression, which suggests that young people and women with depression may have an increased risk of new-onset AF.

**Conclusions and Relevance:**

This study found that depression was associated with a significantly increased cumulative incidence and risk of new-onset AF. Recurrent episodes of depression were associated with even higher risk. These findings suggest the need for adequate screening for AF in people with depression, particularly in younger people and women.

## Key Points

**Question:**

Is depression associated with increased risk of new-onset atrial fibrillation (AF)?

**Findings:**

In this cohort study of 5 031 222 individuals with a follow-up of 43 115 042 person-years, depression was associated with a higher cumulative incidence of new-onset AF. Recurrent episodes of depression were associated with an even higher risk of new-onset AF, and young age and female sex had a significant interaction with depression.

**Meaning:**

Results of this study suggest that depression is associated with an increased risk of new-onset AF, suggesting the need for adequate screening for AF in people with depression.

## Teaser

This cohort study uses data from the Korean National Health Insurance Service database to assess the association of depression with the development of new-onset atrial fibrillation.

## Introduction

A considerable proportion of people are affected by atrial fibrillation (AF), and the prevalence is estimated to grow rapidly because of an aging population.^1,2,3^ In addition to considerable limitations in quality of life, the incidence of major cardiac events, such as ischemic stroke, heart failure, and death, is substantially increased in people with AF.^4,5,6,7^ Recent efforts mainly focus on the prevention of ischemic stroke and treatment of AF through ablation procedures, and progress has been achieved.^4,8,9,10^ However, identification of risk factors for AF and primary prevention of AF have not received as much attention.

It is possible that psychological stress can aggravate or induce all types of tachyarrhythmias through activation of sympathetic tone. Isoproterenol, a sympathomimetic drug, is used in most electrophysiology laboratories to induce paroxysmal supraventricular tachycardia, atrial tachycardia, or AF.^11^ A previous study reported that depression is associated with an increase in sympathetic activity.^12^ Although the study was focused on myocardial remodeling after myocardial infarction, increased sympathetic activity can also have an adverse effect on cardiac rhythm status and might be associated with new-onset AF.^12^ The association between depression and increased risk of cardiovascular events in patients with myocardial infarction is well established.^12,13,14^ However, whether depression is a risk factor for increased risk of new-onset AF remains controversial. In the Trøndelag Health (HUNT) study, the authors found no association between symptoms of anxiety or severe depression and the risk of new-onset AF.^15^ In contrast, researchers from Denmark reported an increased risk of new-onset AF in antidepressant users, especially before initiation of treatment for depression.^16^

Depression is a disease that can be controlled; therefore, evaluation of the association between depression and AF is important from a public health care perspective. We aimed to assess the incidence of new-onset AF in people with and without depression using data from a nationwide health care database.

## Methods

### Nationwide Medical Database

This cohort study obtained data from the Korean National Health Insurance Service (K-NHIS) database. Most Korean people are mandatory subscribers of the K-NHIS, the only medical insurance system available that is managed by the Korean government. Therefore, the K-NHIS database represents the entire population of South Korea, which consists exclusively of East Asian people (Koreans of single race and ethnicity). Furthermore, the K-NHIS offers a regular health checkup for all subscribers, which includes important medical parameters (eg, body weight, height, waist circumference, blood pressure, fasting glucose, lipid profiles, and creatinine level), social habits (eg, alcohol consumption, smoking status, and physical activities), income level, and insurance claims with *International Statistical Classification of Diseases and Related Health Problems, Tenth Revision (ICD-10)* codes. A cohort consisting of people who underwent a nationwide health checkup in a certain year is an important resource for conducting medical research. The nature and characteristics of the K-NHIS database and strongpoints that distinguish the current study from other claim database studies (including availability of various blood tests, such as creatinine level, fasting blood glucose, and lipid profiles; direct measurement of body weight, height, and blood pressure; and surveillance on physical activity, alcohol consumption, and smoking status) have been well described in other studies.^17,18,19^ Use of the K-NHIS database is permitted if the study protocols are approved by both the government’s official review committee and the institutional review board of the relevant medical institution. The institutional review board of Korea University Anam Hospital approved this study and waived the requirement for informed consent because of its retrospective nature. The study followed the Strengthening the Reporting of Observational Studies in Epidemiology (STROBE) reporting guideline.

### Study Population

The cohort used for this study consisted of people who underwent a nationwide health checkup in 2009. The screening period was from January 1, 2002 to December 31, 2008, and baseline medical history was identified during this period. Exclusion criteria included people who (1) were younger than 20 years old, (2) had a history of heart valve surgery, (3) had a previous diagnosis of mitral stenosis, and (4) were diagnosed with AF between January 1, 2002 and December 31,2008. The patients were followed up from January 1, 2009 to December 2018, and the data were analyzed between August 1, 2020 and October 31, 2020.

### Primary Outcome and Definitions

Occurrence of new-onset AF during the follow-up period (from each participant’s health checkup in 2009 to December 31, 2018) was the primary outcome of this study. The incidence of new-onset AF was defined as the number of events calculated per 1000 person-years of follow-up.

A previous diagnosis of depression was defined as the presence of an insurance claim with *ICD-10* codes for depression within 12 months before the 2009 nationwide health checkup. Recurrent depression was defined as the presence of additional claims with *ICD-10* codes for depression during 12 to 24 months after the health checkup in 2009. According to our coding strategy, people with mild forms of depression, such as a single episodes of depression, were not included in the recurrent depression group. Therefore, the recurrent depression group represents people with a more advanced stage of depression. Clinical follow-up was available until December 31, 2018. The exact *ICD-10* codes used in this research are summarized in the eTable in the Supplement. Under the K-NHIS system, prescriptions of selective serotonin reuptake inhibitors, which are the most commonly used antidepressants, are limited to 60 days by law if the prescriber is not a board-certified psychiatrist. Therefore, most patients with depression in South Korea are treated by board-certified psychiatrists. Psychotropic medication, such as anxiolytic drugs (eg, benzodiazepines), and other sedative or hypnotic drugs (eg, zolpidem) can be prescribed by primary care physicians without depression-related *ICD-10* codes. These policies prevent false diagnosis of depression to prescribe anxiolytic or sedative drugs. Identification of new-onset AF was based on the presence of either (1) 2 or more outpatient claims of *ICD-10* codes for AF or (2) 1 or more inpatient claim of *ICD-10* codes for AF.

Review of 1 inpatient record was required for diagnosis of heart failure. Chronic kidney disease was defined as an estimated glomerular filtration rate of <60 mL/min/1.73m^2^. Current smokers were defined as those who smoked 100 or more cigarettes in their life and continued smoking within 1 month before the 2009 nationwide health checkup. Heavy drinkers were defined as those consuming 210 g or more of alcohol per week. Diabetes was diagnosed based on fasting blood glucose (≥126 mg/dL; to convert to mmol/L, multiply by 0.0555) or a claim with *ICD-10* codes for diabetes as diagnosed by a physician. Hypertension was identified based on blood pressure measurement (systolic blood pressure ≥140 mm Hg or diastolic blood pressure ≥90 mm Hg) or by a claim with *ICD-10* codes for hypertension. Regular physical activity was defined as participating in 1 or more high-intensity (eg, running, climbing, or intense bicycle activities) or moderate-intensity (eg, walking fast, tennis, or moderate bicycle activities) physical activity session in a week. The robustness of these definitions has been validated in previous studies.^17,18^

### Statistical Analysis

The *t *test was used to compare continuous variables. Categorical variables were compared with the Fisher exact test or χ^2^ test as appropriate. Unadjusted and adjusted hazard ratios (HRs) and 95% CIs were calculated with Cox proportional hazards regression analysis. Three models were used for multivariable analysis and were adjusted for (1) age and sex; (2) age, sex, body mass index (BMI; calculated as weight in kilograms divided by height in meters squared), smoking status, alcohol consumption status, regular physical activity, income level, diabetes, hypertension, and dyslipidemia; and (3) age, sex, BMI, smoking status, alcohol consumption status, regular physical activity, income level, diabetes, hypertension, dyslipidemia, heart failure, and thyroid disease. The covariates included in the multivariate models were either proven risk factors for AF in prior studies or those reported to have a considerable difference between people with and without depression in this cohort.^3,17,18,19,20^ Because our cohort had nearly 10 years of clinical follow-up data, we treated depression as a time-varying covariate to permit new diagnoses during follow-up. The cumulative incidence of new-onset AF was depicted by Kaplan-Meier curve analysis and compared with the log-rank test. Baseline time was the day of the 2009 nationwide health checkup for all participants in this study for both Cox proportional hazards regression and Kaplan-Meier curve analysis. People were censored if they (1) died, (2) immigrated and were no longer followed up by the K-NHIS, or (3) had new-onset AF (primary outcome of the study). All tests were 2-tailed, and *P* ≤ .05 was considered to be statistically significant. All statistical analyses were performed with SAS, version 9.2 (SAS Institute Inc).

## Results

### Patients

**Figure 1.**
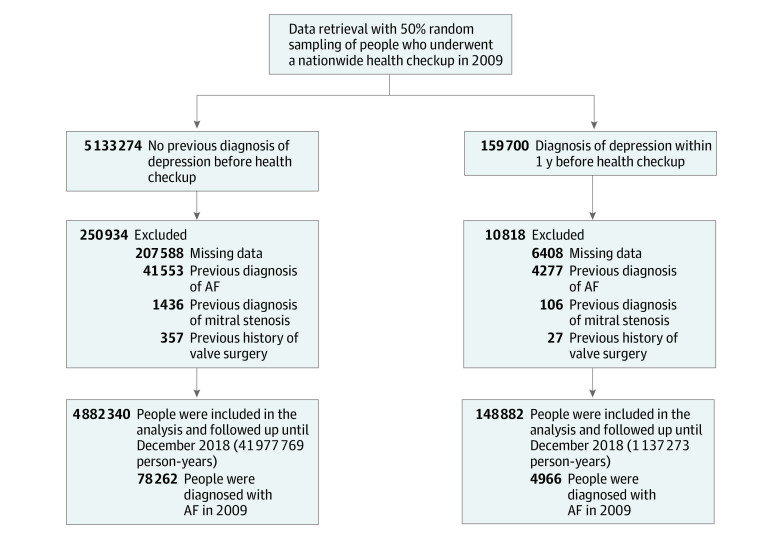
Study Flowchart AF indicates atrial fibrillation.

Data retrieval from the K-NHIS database was done with 50% random sampling among adults in South Korea who underwent a nationwide health checkup in 2009. A total of 5 031 222 individuals with a mean (SD) age of 46.99 (14.06) years (2 771 785 men [55.1%] and 2 259 437 women [44.9%]) were included in the cohort; of these individuals, 148 882 had a previous diagnosis of depression and 4 882 340 did not. The flow of the study is summarized in Figure 1.

Baseline clinical characteristics are summarized in Table 1. The prevalence of diabetes, hypertension, dyslipidemia, and heart failure of the entire cohort was 8.7%, 26.7%, 18.1%, and 0.5%, respectively. People with a previous diagnosis of depression were older than those without such a diagnosis; more likely to be women; and had a higher prevalence of diabetes, hypertension, dyslipidemia, heart failure, schizophrenia, bipolar affective disorders, dementia, alcohol abuse, and thyroid disease. No clinically significant difference was observed in measured BMI, fasting glucose, systolic and diastolic blood pressure, or triglyceride level. Estimated glomerular filtration rates were substantially different, presumably because of age differences.

**Table 1.** Baseline Demographic Characteristics

Characteristic	No. of individuals (%)		*P* value
	Without depression (n = 4 882 340)	With depression (n = 148 882)	
Age, mean (SD)	46.7 (14.0)	56.7 (13.2)	<.001
Age group, y			
20-39	1 581 404 (32.4)	14 221 (9.6)	<.001
40-64	2 702 203 (55.4)	88 965 (59.8)	
≥65	598 733 (12.3)	45 696 (30.7)	
Sex			
Male	2 719 375 (55.7)	52 410 (35.2)	<.001
Female	2 162 965 (44.3)	96 472 (64.8)	
Current smoker	1 305 134 (26.7)	22 042 (14.8)	<.001
Alcohol consumption			
None or mild to moderate	4 487 020 (91.9)	142 434 (95.7)	<.001
Heavy drinker	395 320 (8.1)	6448 (4.3)	
Regular exercise	883 371 (18.1)	28 027 (18.8)	<.001
Income quartile^a^			
Q1	1 046 466 (21.4)	31 337 (21.1)	<.001
Q2	1 099 965 (22.5)	28 202 (18.9)	
Q3	1 311 375 (26.9)	37 064 (24.9)	
Q4	1 424 534 (29.2)	52 279 (35.1)	
Diabetes	413 233 (8.5)	22 260 (15.0)	<.001
Hypertension	1 276 098 (26.1)	65 932 (44.3)	<.001
Dyslipidemia	862 379 (17.7)	46 647 (31.3)	<.001
Heart failure	23 440 (0.5)	2578 (1.7)	<.001
Schizophrenia	6904 (0.1)	2579 (1.7)	<.001
Bipolar affective disorder	4757 (0.1)	3168 (2.1)	<.001
Dementia	9314 (0.2)	2744 (1.8)	<.001
Alcohol abuse	948 (0.02)	449 (0.3)	<.001
Hypothyroidism or hyperthyroidism	151 581 (3.1)	12 765 (8.6)	<.001
Fasting glucose, mean (SD), mg/dL	97.1 (23.7)	100.2 (26.4)	<.001
Body mass index, mean (SD)^b^	23.7 (3.2)	23.9 (3.2)	<.001
Waist circumference, cm, mean (SD)	80.2 (9.5)	80.9 (9.0)	<.001
Blood pressure, mm Hg, mean (SD)			
Systolic	122.4 (15.0)	123.6 (15.6)	<.001
Diastolic	76.3 (10.0)	76.4 (10.0)	.07
eGFR, mean (SD)	87.6 (28.1)	75.2 (27.0)	<.001
Triglyceride, mg/dL (IQR)	113.3 (113.3-113.3)	116.3 (115.9-116.6)	<.001

Abbreviations: eGFR, estimated glomerular filtration rate; Q, quartile.

SI conversion factor: To convert fasting glucose to mmol/L, mutiply by 0.0555; to convert triglyceride levels to mmol/L, multiply by 0.0113.

^a^ Q1 represents the lowest income, and Q4 represents the highest.

^b^ Calculated as weight in kilograms divided by height in meters squared.

### New-Onset AF

**Figure 2.**
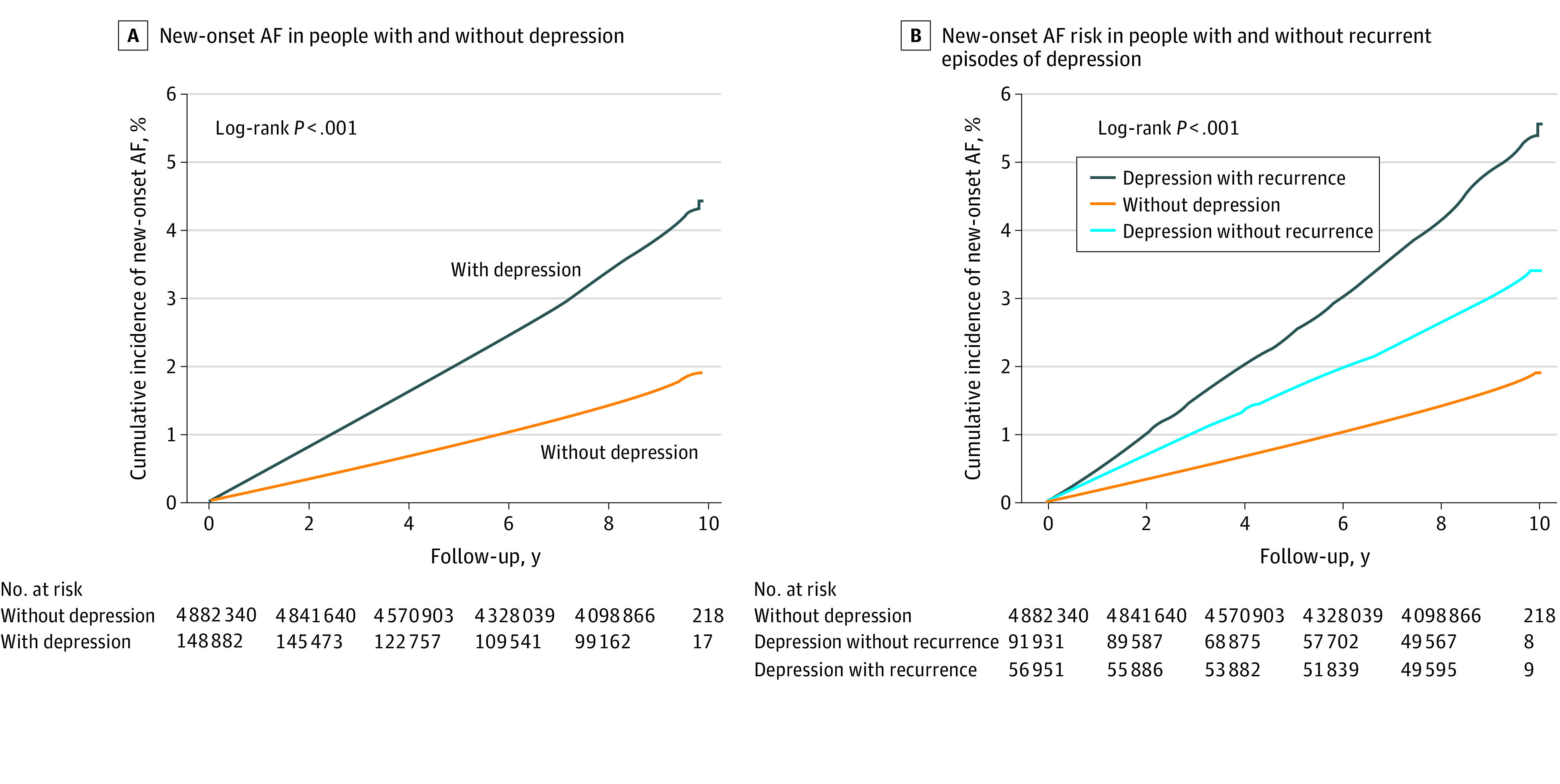
Risk of New-Onset Atrial Fibrillation (AF) Associated With Depression A, The risk of new-onset AF was substantially higher in people with a previous diagnosis of depression. B, People with recurrent episodes of depression had the highest risk of new-onset AF.

We retrieved the follow-up clinical data until December 31, 2018, which included 1 137 273 person-years of follow-up for those with depression and 41 977 769 person-years follow-up for those without depression. In 4 882 340 people without depression, 78 262 (1.6%) were diagnosed with new-onset AF during the follow-up period (incidence, 1.86 per 1000 person-years). The risk of new-onset AF was significantly higher in people with a previous diagnosis of depression (HR, 2.36; 95% CI, 2.30-2.43; *P* < .001), and the incidence of new-onset AF was 4.37 per 1000 person-years (4966 new-onset AF cases among 148 882) (Table 2). After multivariable adjustment for age, sex, BMI, smoking status, alcohol consumption status, regular physical activity, income level, diabetes mellitus, hypertension, dyslipidemia, heart failure, and thyroid disease, a previous diagnosis of depression was associated with a 25.1% increased risk of new-onset AF (HR, 1.25; 95% CI, 1.22-1.29; *P* < .001) (Table 2). When depression was included in the model as a time-varying covariate, the risk of new-onset AF was increased by 33.1% in the depression group (Table 2). The Kaplan-Meier curve analysis showed significantly higher cumulative incidence of new-onset AF in people with depression (cumulative incidence, 4.44% vs 1.92%; log-rank *P* < .001; Figure 2).

**Table 2.** Multivariate Cox Proportional Hazards Regression Analysis of the Association of Depression With Risk of AF

	No. of individuals	New-onset AF	Follow-up, person-years	Incidence	Unadjusted, HR (95% CI)	Model 1, HR (95% CI)^**a**^	Model 2, HR (95% CI)^**b**^	Model 3, HR (95% CI)^**c**^	Model 4, HR (95% CI)^**d**^	Model 5, HR (95% CI)^**e**^
Depression										
No	4 882 340	78 262	41 977 769	1.86	1 [Reference]	1 [Reference]	1 [Reference]	1 [Reference]	1 [Reference]	1 [Reference]
Yes	148 882	4966	1 137 273	4.37	2.36 (2.30-2.43)	1.34 (1.30-1.38)	1.30 (1.26-1.34)	1.25 (1.22-1.29)	1.25 (1.21-1.29)	1.33 (1.30-1.36)
Without recurrent episode	91 931	2191	636 896	3.44	1.88 (1.81-1.97)	1.23 (1.18-1.29)	1.21 (1.16-1.26)	1.17 (1.12-1.22)	1.17 (1.12-1.22)	1.28 (1.24-1.32)
With recurrent episode	56 951	2775	500 377	5.55	2.96 (2.85-3.07)	1.43 (1.38-1.49)	1.38 (1.33-1.44)	1.32 (1.27-1.37)	1.32 (1.27-1.37)	1.38 (1.34-1.42)

Abbreviations: AF, atrial fibrillation; HR, hazard ratio.

^a^ Adjusted for age and sex.

^b^ Model 2: Model 1 plus adjustment for body mass index (calculated as weight in kilograms divided by height in meters squared), smoking status, alcohol consumption status, regular physical activity, income level, diabetes mellitus, hypertension, and dyslipidemia.

^c^ Model 3: Model 2 plus adjustment for heart failure and thyroid disease.

^d^ Model 4: the same adjustment as in Model 3 except that age was taken as a time scale truncated on the left at age in 2009 (study entry).

^e^ Model 5: the same adjustment as in Model 3 plus depression as a time-varying covariate.

### Recurrent Episodes of Depression

Among 148 882 people with depression, 56 951 people had recurrent episodes of depression. The incidence of new-onset AF in people with recurrent depression (incidence, 5.55 per 1000 person-years) was significantly higher as compared with people without recurrent episodes of depression (incidence, 3.44 per 1000 person-years) and people with no depression (incidence, 1.86 per 1000 person-years) (Table 2). After multivariable adjustment, people with recurrent episodes of depression had a 32.2% (HR, 1.32; 95% CI, 1.27-1.37; *P* < .001) increased risk of new-onset AF (37.8% [HR, 1.38; 95% CI, 1.34-1.42; *P* < .001] increased risk if depression was included in the model as a time-varying covariate) compared with people without depression (Table 2 and Figure 2). Compared with people with depression but without recurrent episodes, the risk of new-onset AF was significantly higher in people with recurrent episodes (cumulative incidence, 5.56% vs 3.43%; log-rank *P* < .001) (Table 2 and Figure 2).

### Subgroup Analysis

A significant interaction was observed between depression and age with respect to the risk of new-onset AF. People aged 20 to 39 years had a 58.3% increased risk of new-onset AF associated with depression (adjusted HR, 1.58; 95% CI, 1.24-2.02) (Table 3). In contrast, people 65 years and older with depression had only a 16.8% increased risk of new-onset AF (adjusted HR, 1.17; 95% CI, 1.13-1.21; *P* for interaction <.001). In addition, a significant interaction was observed between depression and female sex with regard to the risk of new-onset AF. The risk of new-onset AF increased by 31.5% in women with depression (adjusted HR, 1.32; 95% CI, 1.27-1.37) and by 17.0% in men with depression (adjusted HR, 1.17; 95% CI, 1.12-1.22) (Table 3).

**Table 3.** Subgroup Analysis

Characteristic	Hazard ratio (95% CI)^**a**^	*P* value for interaction
Age group, y		
20-39	1.58 (1.24-2.02)	<.001
40-64	1.39 (1.33-1.46)	
65 and older	1.17 (1.13-1.21)	
Sex		
Male	1.17 (1.12-1.22)	<.001
Female	1.32 (1.27-1.37)	
Heavy drinker		
No	1.25 (1.22-1.29)	.64
Yes	1.22 (1.07-1.40)	
Regular exercise		
No	1.24 (1.20-1.28)	.38
Yes	1.31 (1.23-1.40)	
Current smoker		
No	1.25 (1.22-1.29)	.43
Yes	1.24 (1.15-1.35)	
eGFR		
<30	1.29 (1.10-1.52)	.34
30-59	1.24 (1.19-1.30)	
60-90	1.23 (1.18-1.29)	
>90	1.31 (1.21-1.42)	
Obesity		
No (BMI <25)	1.26 (1.21-1.31)	.11
Yes (BMI ≥25)	1.24 (1.19-1.30)	
Abdominal obesity		
No	1.27 (1.22-1.32)	<.001
Yes	1.22 (1.16-1.28)	
Diabetes		
No	1.24 (1.20-1.28)	.71
Yes	1.31 (1.23-1.39)	
Dyslipidemia		
No	1.24 (1.20-1.29)	.62
Yes	1.28 (1.22-1.34)	
Hypertension		
No	1.31 (1.24-1.38)	.002
Yes	1.23 (1.19-1.27)	

Abbreviations: BMI, body mass index (calculated as weight in kilograms divided by height in meters squared); eGFR, estimated glomerular filtration rate.

^a^ Hazard ratios were adjusted for age, sex, BMI, smoking status, alcohol consumption status, regular physical activity, income level, diabetes mellitus, hypertension, dyslipidemia, heart failure, and thyroid disease.

## Discussion

In this cohort study, a previous diagnosis of depression was associated with a significantly increased risk of new-onset AF (adjusted HR, 1.25; 95% CI, 1.22-1.29), and people with recurrent episodes of depression were subject to an even higher risk of new-onset AF (adjusted HR, 1.32; 95% CI, 1.27-1.37). In addition, significant interactions were found between young age and depression and female sex and depression with respect to new-onset AF. Previous studies examining the association between depression and new-onset AF have reported conflicting results.^15,16,21^ In this study, we found an association between previous diagnosis of depression and increased risk of new-onset AF by analyzing nationwide medical data with a large sample size (5 031 222 people) and long follow-up duration (43 115 042 person-years).

### Depression and AF

In people with underlying AF, the prevalence of depression is estimated to be 8% to 38%,^22,23,24,25^ which is higher than the 1% to 2% in the general population.^26,27^ It is relatively well established that people with AF are at increased risk of developing depression. However, whether depression can provoke development of new-onset AF remains to be elucidated. A prospective cohort study consisting of 30 746 women without a history of cardiovascular disease found no significant association between global psychological distress and specific depressive symptoms and development of new-onset AF.^21^ The HUNT study, which included 37 402 adults in Norway, also found no significant association between depression and new-onset AF.^15^ In contrast, a cohort of 6644 people from the United States revealed a 34% increased risk of new-onset AF.^28^ A nationwide registry-based study from Denmark revealed that use of antidepressants was associated with an increased risk of new-onset AF, particularly before the initiation of treatment for depression.^16^ However, this association was gradually attenuated during the following year.^16^ An association has been found between depression and clinical outcomes and response to treatment in patients with AF. A previous study reported that depressive symptoms were associated with increased cardiovascular mortality in patients with comorbid AF and heart failure who received optimized treatment.^29^ Depression was a major risk factor for recurrence of AF after electrical cardioversion, suggesting an association between depression and AF pathogenesis.^30^

The present study, which had a large sample size, revealed that a previous diagnosis of depression was associated with a significantly increased risk of new-onset AF. In contrast with the study from Denmark,^16^ the increased risk of new-onset AF in people with depression continued during the 10-year follow-up period, with progressive divergence observed in the cumulative incidence of new-onset AF between people with and without depression. Another important finding of this study was that people with recurrent episodes of depression were at even higher risk of new-onset AF, suggesting a semiquantitative association between depression and new-onset AF. We had a sufficient screening period, from 2002 to 2008, and any individual with an insurance claim with *ICD-10* codes for AF during the period was identified and excluded from the analysis. Therefore, we were able to eliminate the potential association of AF with depression to measure only the association between depression and AF. Furthermore, because virtually all Korean individuals are mandatory subscribers of the K-NHIS, it is unlikely that symptomatic new-onset AF events remained undetected during follow-up.

### Age and Sex

This study revealed that young age and female sex had significant interactions with the association between depression and new-onset AF. The underlying mechanism for this interaction is not clear. Previous studies reported that depression can augment sympathetic activity,^12,31^ which can in turn increase the risk of new-onset AF. Young people might also have an increased risk for emotional stress and anxiety, which can contribute to an increased risk for various types of arrhythmias.^32^ Whether the elevated sympathetic activity and emotional stress adversely affect young people and women remains to be elucidated. A previous study suggested that nongenetic risk factors for AF, such as body weight, alcohol, hypertension, and diabetes, are equally important in both young and older people.^3^ In the study, significant interactions were observed between age and hypertension and diabetes mellitus, with young people being more susceptible, a similar phenomenon observed in the current study with regard to depression. Whether treatment of depression in young people can prevent occurrence of new-onset AF will be an important area of future research.

Increased risk of new-onset AF in women with depression requires further research. Atrial fibrillation in women has different characteristics than AF in men with respect to response to treatment, stroke risk, and clinical outcomes.^33,34^ However, the difference in underlying pathogenesis of AF in women and men has not been fully elucidated. A previous report suggested that estrogen can have a protective benefit against AF, which was shown by substantially less shortening of the effective refractory period in response to rapid pacing in premenopausal women than postmenopausal women or men.^35^ In turn, fluctuation in estrogen levels in women is one of the proposed mechanisms of a higher incidence of depression in women.^36^ Lei et al^37^ reported that estrogen levels were substantially lower in patients with recurrent depression compared with patients with first-episode depression. Whether disrupted homeostasis of estrogen in women with depression is associated with increased risk for AF development remains an area for future research.

### Strengths and Limitations

A strength of this study is that the results of various blood tests, including creatinine level, fasting blood glucose, and lipid profiles; direct measurement of body weight, height, and blood pressure; and surveillance on physical activity, alcohol consumption, and smoking status, were provided by the nationwide health checkup. This factor distinguishes our study from other claim-databased studies. This study has limitations. First, our study was based on insurance claims with *ICD-10* codes for depression and AF. Although the identification of AF was not based on the interpretation of an electrocardiogram, the claim-based identification strategy of AF is well established.^3,17,18,19^ Second, additional clinical information, such as type of AF and size of left atrium, were not available. Third, the possibility of undetected confounders, such as use of antidepressants, diameter of left atrium, or left ventricular function, is another limitation of this study. Fourth, the K-NHIS database exclusively consists of East Asian people, and the results from this study cannot be generalized to other racial and ethnic groups. Fifth, recurrent depression as defined in this study represents a severe form of depression, but we could not distinguish repetitive episodes of depression from ongoing depression.

## Conclusion

Based on data obtained from a Korean nationwide health checkup cohort, this cohort study found that depression was associated with a significantly increased risk of new-onset AF after adjusting for various covariates. An exposure-response association was observed, with recurrent episodes of depression associated with an even higher risk of new-onset AF. Young age and female sex were also found to have a significant interaction in the association between depression and new-onset AF. Whether adequate treatment of depression can reduce the risk of new-onset AF needs further examination.

## Notes

Supplement. **eTable.** Diagnostic Codes

## References

[1] Kjerpeseth LJ, Igland J, Selmer R, . Prevalence and incidence rates of atrial fibrillation in Norway 2004-2014. Heart. 2021;107(3):201-207. doi:10.1136/heartjnl-2020-316624 32820014 PMC7815897

[2] Dai H, Zhang Q, Much AA, . Global, regional, and national prevalence, incidence, mortality, and risk factors for atrial fibrillation, 1990-2017: results from the Global Burden of Disease Study 2017. Eur Heart J Qual Care Clin Outcomes. 2021;7(6):574-582. doi:10.1093/ehjqcco/qcaa061 32735316 PMC8557444

[3] Kim YG, Han KD, Choi JI, . Non-genetic risk factors for atrial fibrillation are equally important in both young and old age: a nationwide population-based study. Eur J Prev Cardiol. 2021;28(6):666-676. doi:10.1177/2047487320915664 34021574

[4] Marrouche NF, Brachmann J, Andresen D, ; CASTLE-AF Investigators. Catheter ablation for atrial fibrillation with heart failure. N Engl J Med. 2018;378(5):417-427. doi:10.1056/NEJMoa1707855 29385358

[5] Prabhu S, Taylor AJ, Costello BT, . Catheter ablation versus medical rate control in atrial fibrillation and systolic dysfunction: the CAMERA-MRI study. J Am Coll Cardiol. 2017;70(16):1949-1961. doi:10.1016/j.jacc.2017.08.041 28855115

[6] Freedman B, Potpara TS, Lip GY. Stroke prevention in atrial fibrillation. Lancet. 2016;388(10046):806-817. doi:10.1016/S0140-6736(16)31257-0 27560276

[7] Kim YG, Shim J, Choi JI, Kim YH. Radiofrequency catheter ablation improves the quality of life measured with a short form-36 questionnaire in atrial fibrillation patients: a systematic review and meta-analysis. PLoS One. 2016;11(9):e0163755. doi:10.1371/journal.pone.0163755 27681507 PMC5040266

[8] Packer DL, Mark DB, Robb RA, ; CABANA Investigators. Effect of catheter ablation vs antiarrhythmic drug therapy on mortality, stroke, bleeding, and cardiac arrest among patients with atrial fibrillation: the CABANA randomized clinical trial. JAMA. 2019;321(13):1261-1274. doi:10.1001/jama.2019.0693 30874766 PMC6450284

[9] Andrade JG, Wells GA, Deyell MW, ; EARLY-AF Investigators. Cryoablation or drug therapy for initial treatment of atrial fibrillation. N Engl J Med. 2021;384(4):305-315. doi:10.1056/NEJMoa2029980 33197159

[10] Okumura K, Akao M, Yoshida T, ; ELDERCARE-AF Committees and Investigators. Low-dose edoxaban in very elderly patients with atrial fibrillation. N Engl J Med. 2020;383(18):1735-1745. doi:10.1056/NEJMoa2012883 32865374

[11] Kim YG, Han S, Choi JI, . Impact of persistent left superior vena cava on radiofrequency catheter ablation in patients with atrial fibrillation. Europace. 2019;21(12):1824-1832. doi:10.1093/europace/euz254 31578551

[12] Shi S, Liang J, Liu T, . Depression increases sympathetic activity and exacerbates myocardial remodeling after myocardial infarction: evidence from an animal experiment. PLoS One. 2014;9(7):e101734. doi:10.1371/journal.pone.0101734 25036781 PMC4103791

[13] Meijer A, Conradi HJ, Bos EH, . Adjusted prognostic association of depression following myocardial infarction with mortality and cardiovascular events: individual patient data meta-analysis. Br J Psychiatry. 2013;203(2):90-102. doi:10.1192/bjp.bp.112.111195 23908341

[14] Huffman JC. Review: depression after myocardial infarction is associated with increased risk of all-cause mortality and cardiovascular events. Evid Based Ment Health. 2013;16(4):110. doi:10.1136/eb-2013-101537 24091616

[15] Feng T, Malmo V, Laugsand LE, . Symptoms of anxiety and depression and risk of atrial fibrillation-the HUNT study. Int J Cardiol. 2020;306:95-100. doi:10.1016/j.ijcard.2019.11.107 31759687

[16] Fenger-Grøn M, Vestergaard M, Pedersen HS, . Depression, antidepressants, and the risk of non-valvular atrial fibrillation: a nationwide Danish matched cohort study. Eur J Prev Cardiol. 2019;26(2):187-195. doi:10.1177/2047487318811184 30452291

[17] Kim YG, Han KD, Choi JI, . Frequent drinking is a more important risk factor for new-onset atrial fibrillation than binge drinking: a nationwide population-based study. Europace. 2020;22(2):216-224. doi:10.1093/europace/euz256 31620800

[18] Kim YG, Han KD, Choi JI, . Impact of the duration and degree of hypertension and body weight on new-onset atrial fibrillation: a nationwide population-based study. Hypertension. 2019;74(5):e45-e51. doi:10.1161/HYPERTENSIONAHA.119.13672 31522617

[19] Kim YG, Han KD, Choi JI, . The impact of body weight and diabetes on new-onset atrial fibrillation: a nationwide population based study. Cardiovasc Diabetol. 2019;18(1):128. doi:10.1186/s12933-019-0932-z 31575379 PMC6774211

[20] Benjamin EJ, Levy D, Vaziri SM, D’Agostino RB, Belanger AJ, Wolf PA. Independent risk factors for atrial fibrillation in a population-based cohort: the Framingham Heart Study. JAMA. 1994;271(11):840-844. doi:10.1001/jama.1994.03510350050036 8114238

[21] Whang W, Davidson KW, Conen D, Tedrow UB, Everett BM, Albert CM. Global psychological distress and risk of atrial fibrillation among women: the Women’s Health Study. J Am Heart Assoc. 2012;1(3):e001107. doi:10.1161/JAHA.112.001107 23130138 PMC3487320

[22] von Eisenhart Rothe AF, Goette A, Kirchhof P, . Depression in paroxysmal and persistent atrial fibrillation patients: a cross-sectional comparison of patients enroled in two large clinical trials. Europace. 2014;16(6):812-819. doi:10.1093/europace/eut361 24351885

[23] Dąbrowski R, Smolis-Bąk E, Kowalik I, Kazimierska B, Wójcicka M, Szwed H. Quality of life and depression in patients with different patterns of atrial fibrillation. Kardiol Pol. 2010;68(10):1133-1139. 20967710

[24] Schnabel RB, Michal M, Wilde S, . Depression in atrial fibrillation in the general population. PLoS One. 2013;8(12):e79109. doi:10.1371/journal.pone.0079109 24324579 PMC3850915

[25] Thrall G, Lip GY, Carroll D, Lane D. Depression, anxiety, and quality of life in patients with atrial fibrillation. Chest. 2007;132(4):1259-1264. doi:10.1378/chest.07-0036 17646231

[26] Glaesmer H, Riedel-Heller S, Braehler E, Spangenberg L, Luppa M. Age- and gender-specific prevalence and risk factors for depressive symptoms in the elderly: a population-based study. Int Psychogeriatr. 2011;23(8):1294-1300. doi:10.1017/S1041610211000780 21729425

[27] Olsen LR, Mortensen EL, Bech P. Prevalence of major depression and stress indicators in the Danish general population. Acta Psychiatr Scand. 2004;109(2):96-103. doi:10.1046/j.0001-690X.2003.00231.x 14725589

[28] Garg PK, O’Neal WT, Diez-Roux AV, Alonso A, Soliman EZ, Heckbert S. Negative affect and risk of atrial fibrillation: MESA. J Am Heart Assoc. 2019;8(1):e010603. doi:10.1161/JAHA.118.010603 30563392 PMC6405728

[29] Frasure-Smith N, Lespérance F, Habra M, ; Atrial Fibrillation and Congestive Heart Failure Investigators. Elevated depression symptoms predict long-term cardiovascular mortality in patients with atrial fibrillation and heart failure. Circulation. 2009;120(2):134-140. doi:10.1161/CIRCULATIONAHA.109.851675 19564557

[30] Lange HW, Herrmann-Lingen C. Depressive symptoms predict recurrence of atrial fibrillation after cardioversion. J Psychosom Res. 2007;63(5):509-513. doi:10.1016/j.jpsychores.2007.07.010 17980224

[31] Barton DA, Dawood T, Lambert EA, . Sympathetic activity in major depressive disorder: identifying those at increased cardiac risk? J Hypertens. 2007;25(10):2117-2124. doi:10.1097/HJH.0b013e32829baae7 17885556

[32] Ziegelstein RC. Acute emotional stress and cardiac arrhythmias. JAMA. 2007;298(3):324-329. doi:10.1001/jama.298.3.324 17635893

[33] Volgman AS, Benjamin EJ, Curtis AB, ; American College of Cardiology Committee on Cardiovascular Disease in Women. Women and atrial fibrillation. J Cardiovasc Electrophysiol. 2021;32(10):2793-2807. doi:10.1111/jce.14838 33332669 PMC8281363

[34] Westerman S, Wenger N. Gender differences in atrial fibrillation: a review of epidemiology, management, and outcomes. Curr Cardiol Rev. 2019;15(2):136-144. doi:10.2174/1573403X15666181205110624 30516110 PMC6520576

[35] Tse HF, Oral H, Pelosi F, Knight BP, Strickberger SA, Morady F. Effect of gender on atrial electrophysiologic changes induced by rapid atrial pacing and elevation of atrial pressure. J Cardiovasc Electrophysiol. 2001;12(9):986-989. doi:10.1046/j.1540-8167.2001.00986.x 11573707

[36] Shors TJ, Leuner B. Estrogen-mediated effects on depression and memory formation in females. J Affect Disord. 2003;74(1):85-96. doi:10.1016/S0165-0327(02)00428-7 12646301 PMC3374589

[37] Lei R, Sun Y, Liao J, . Sex hormone levels in females of different ages suffering from depression. BMC Womens Health. 2021;21(1):215. doi:10.1186/s12905-021-01350-0 34022874 PMC8141202

