# Supplementary material for: Association of Depression With Atrial Fibrillation in South Korean Adults
Source: JAMA Netw Open. 2022 Jan 4;5(1):e2141772. doi: 10.1001/jamanetworkopen.2021.41772 (PMC8728611; doi:10.1001/jamanetworkopen.2021.41772)
Supplement: Supplement. — eTable. Diagnostic Codes [file jamanetwopen-e2141772-s001.pdf]

Supplementary Online Content

Kim YG, Lee KN, Han KD, et al. Association of depression with atrial fibrillation in South Korean adults. *JAMA Netw Open*. 2022;5(1):e2141772. doi:10.1001/jamanetworkopen.2021.41772

eTable. Diagnostic codes.

This supplementary material has been provided by the authors to give readers additional information about their work.

©2022 Kim YG, et al. *JAMA Network Open*.

eTable. Diagnostic Codes.

	ICD-10 codes
**Depression**	F32 (all sub-codes), F33 (all sub-codes)
**Atrial fibrillation**	I48 (all sub-codes)
**Mitral stenosis**	I05.0, I05.2, I34.2
**Valve surgery**	O1781, O1791, O1782, O1792, O1783, O1793, O1799, O1797
**Heart failure**	I50 (all sub-codes)
**Atrial fibrillation**	I48 (all sub-codes)
**Type 2 diabetes**	E11 – E14 (all sub-codes)
**Hypertension**	I10 – I13, I15 (all sub-codes)
**Dyslipidemia**	I78 (all sub-codes)
**Chronic kidney disease**	Based on creatinine checked during national health check-up
**Schizophrenia**	F20 (all sub-codes)
**Bipolar affective disorder**	F31 (all sub-codes)
**Dementia**	G30 (all sub-codes), F02 (all sub-codes), F03 (all sub-codes)
**Hypo- or hyperthyroidism**	E03 (all sub-codes), E05 (all sub-codes)

©2022 Kim YG, et al. *JAMA Network Open*.
